# Independent neural drives and distinct motor unit discharge characteristics in hamstring muscles during isometric knee flexion

**DOI:** 10.1007/s00421-025-05953-5

**Published:** 2025-09-04

**Authors:** Chrysostomos Sahinis, Ioannis G. Amiridis, Dario Farina, Roger M. Enoka, Eleftherios Kellis

**Affiliations:** 1 Laboratory of Neuromechanics, Department of Physical Education and Sport Sciences at Serres, Aristotle University of Thessaloniki, Agios Ioannis, 62110 Serres, Greece; 2 Department of Bioengineering, Imperial College London, London, UK; 3 Department of Integrative Physiology, University of Colorado, Boulder, CO USA

**Keywords:** Hamstrings, Semitendinosus, Biceps femoris, Motor unit, Muscle length

## Abstract

**Purpose:**

Our study investigated the discharge characteristics of motor units (MUs) in the semitendinosus (ST) and biceps femoris (BF) at three knee-joint angles that varied muscle length.

**Methods:**

Fifteen males (21.1 ± 2.8 years) performed steady isometric contractions with the knee flexors at four target torques (10%, 20%, 40%, and 60% of maximal voluntary contraction, MVC) at each of the three knee-joint angles (0°: long, 45°: intermediate, and 90°: short length). High-density electromyographic signals were recorded and decomposed into MU discharge times. We calculated mean discharge rate (MDR), the coefficient of variation for interspike interval (CoV ISI), and the standard deviation of the filtered cumulative spike train (SD of fCST). In addition, the neural drive within and between muscles was estimated from the cross-correlation of the fCST.

**Results:**

Analysis of variance indicated that MVC was greatest at the long length and torque steadiness was worst at the intermediate length (*p* < 0.05). Linear mixed models revealed that BF exhibited greater MDR and variability in neural drive (SD of fCST), whereas the MUs in ST displayed greater discharge rate variability (CoV ISI) (*p* < 0.05). Cross-correlation of the estimated neural drives to ST and BF was relatively low, suggesting independent neural control of the two muscles. Moreover, the variability in neural drive for ST was more strongly correlated with torque steadiness (CoV torque) than that for BF.

**Conclusion:**

The findings indicate that MU discharge characteristics differed for ST and BF across knee-joint angles, with each muscle receiving a distinct neural drive highlighting the importance of muscle-specific training strategies.

**Supplementary Information:**

The online version contains supplementary material available at 10.1007/s00421-025-05953-5.

## Introduction

The hamstring muscle group includes the biarticular semimembranosus, semitendinosus (ST), and long head of the biceps femoris (BF), as well as the mono-articular short head of biceps femoris. There are significant differences in muscle architecture (Kellis et al. 2012; Kellis 2016, 2018) and tendon mechanics (Sahinis et al. 2021; Kellis and Sahinis 2022; Sahinis and Kellis 2024a) that likely influence the contribution of each muscle to the net force and the muscle–tendon unit strain experienced during vigorous exercise, such as sprinting and stretching (Hoskins and Pollard 2005; Malliaropoulos et al. 2010).

The control of muscle force relies on motor neurons being activated by a combination of excitatory and inhibitory synaptic inputs (Heckman and Enoka 2012; Enoka and Duchateau 2017). These inputs are distributed to sets of motor neurons and shared among many motor neurons innervating a muscle (Laine et al. 2015; Negro et al. 2016b). The ensemble of action potentials discharged by these motor neurons is known as the neural drive and controls the force generated by the muscle (Negro et al. 2009; Farina and Negro 2015). The shared inputs originate from such sources as descending pathways and afferent axons from the periphery (Lemon 2008; Heckman and Enoka 2012; Glover and Baker 2022).

Recent findings on the existence of multiple neural modules within a muscle, such as the vastii (Del Vecchio et al. 2023) and plantar flexors (Levine et al. 2023; Weinman et al. 2024), suggests that the shared synaptic input is not distributed uniformly among motor neurons innervating individual or synergistic muscles (Laine et al. 2015; Avrillon et al. 2021). The presence of multiple neural modules provides the foundation for more dynamic and adaptable system that can be adjusted to meet functional demands and task-specific requirements (Farina and Negro 2015; Hug et al. 2023). However, the distribution of shared synaptic inputs in the hamstrings, specifically between the ST and BF, has never been explored.

Previous work using muscle functional magnetic resonance imaging (Schuermans et al. 2014; Tampere et al. 2021) and surface electromyography (sEMG) (Kellis and Katis 2008; Hegyi et al. 2019) has revealed significant functional differences between ST and BF. Furthermore, intramuscular EMG studies have found lower motor unit (MU) discharge rates in the BF relative to the medial hamstrings (Kirk and Rice 2017; Kirk et al. 2018). Although measures of transverse relaxation times and sEMG amplitude provide indirect measures of activation among the hamstring muscles, they cannot provide information on the level of common synaptic input (Le Rumeur et al. 1994; Saab et al. 1999; Farina and Enoka 2023). Alternatively, high-density surface EMG (HD-sEMG) provides an approach that can investigate differences in neural drive and synaptic input between these two muscles (Laine et al. 2015; Avrillon et al. 2021; Del Vecchio et al. 2023; Levine et al. 2023; Sahinis et al. 2024; Weinman et al. 2024).

In addition, the relations between joint angle, muscle length, and neural activation in the hamstrings remain unclear (Kellis and Blazevich 2022). Joint angle influences muscle length (Gordon et al. 1966), which alters contraction force and twitch characteristics (Marsh et al. 1981; Bigland-Ritchie et al. 1992). However, evidence on how these factors influence the discharge characteristics of MUs is mixed. For example, intramuscular recordings performed by Kirk and Rice (2017) indicated reduced discharge rates at longer muscle lengths, whereas HD-sEMG recordings by Sahinis et al. (2024) found no significant changes in discharge characteristics for MUs in ST across knee angles. These discrepancies may be attributed to differences in EMG methodology and joint configuration during assessment.

The purpose of our study was to compare MU discharge characteristics between the ST and BF muscles across three knee-joint angles. We hypothesized that weak levels of shared synaptic input between ST and BF would result in relatively independent control of each muscle. The hypothesis is based on the differences in anatomy between ST and BF (Kellis 2018; Kellis and Blazevich 2022); BF has a greater cross-sectional area and pennation angle and is designed for force generation, whereas the ST has a longer fascicle-to-muscle length ratio that enables greater excursion capacity (Kellis 2018; Kellis and Blazevich 2022). Demonstrating this independence may enhance therapeutic interventions for conditions often compromised after hamstring injuries or anterior cruciate ligament reconstruction.

## Methods

### Participants

Fifteen healthy males were enrolled in the study (age: 21.1 ± 2.8 years; height: 1.83 ± 0.77 m; body mass: 85.2 ± 7.6 kg). The participants were limited to males because preliminary tests indicated that it was difficult to identify a sufficient number of MUs in the hamstring muscles of females (Sahinis et al. 2024).

Exclusion criteria included any history of lower limb injuries, knee surgeries, or neurological disorders, such as hamstring strains or other muscle and ligament-related knee injuries in the previous 12 months. The participants were physically active (1693 ± 699 Metabolic Equivalent Task-minutes/week), but they were not involved in a specific sport or exercise regimen during the study period. The study protocol adhered to the Declaration of Helsinki and received approval from the Ethics Committee of the Aristotle University of Thessaloniki (ERC-005/2022). Written informed consent was obtained from all participants before commencing the experiment.

### Experimental setup

**Fig. 1:**
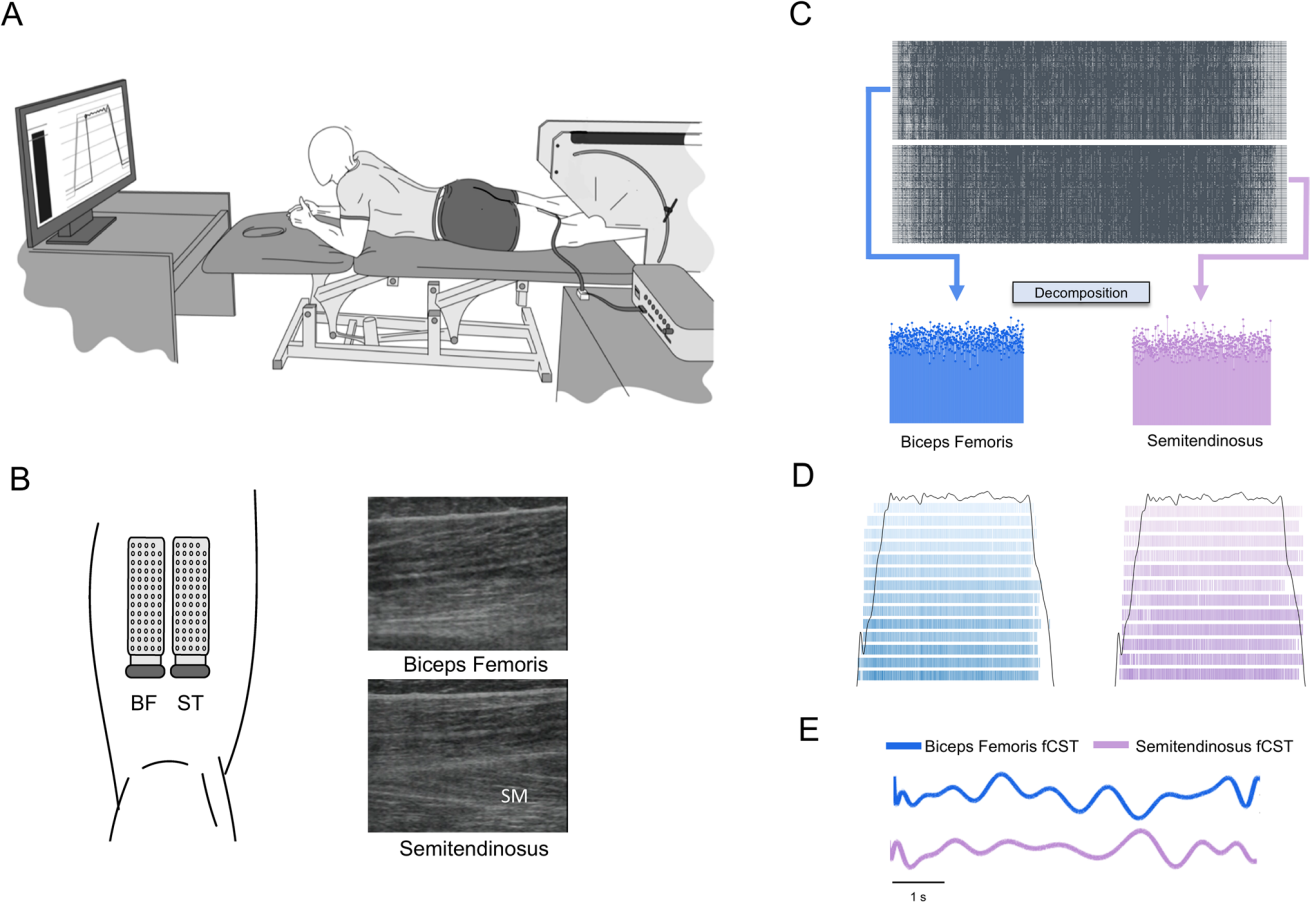
**A** Experimental setup. HD-sEMG signals were recorded from the semitendinosus (ST) and biceps femoris (BF) muscles of the left leg. Visual feedback of the target and applied torques was provided during the ramp-up, plateau, and ramp-down phases (monitor) on a moment-to-moment basis (left side of monitor). The display covered ~ 80% of the screen. **B** Electrode placement and representative longitudinal ultrasound scans of the biceps femoris (BF, upper image) and semitendinosus (ST, lower image). Note that the ST is superficial to the semimembranosus (SM), confirming the anatomical distinction at the site of electrode placement. **C** Motor unit identification through decomposition of HD-sEMG signals from the ST and BF during knee flexion task at 20% of MVC torque. **D** Raster plots displaying the spike trains of the identified motor units from the BF (left panel) and ST (right panel) with the superimposed torque trace (black line) during a trapezoidal contraction at 20% of MVC torque. **E** Representative filtered cumulative spike train (fCST) during the 6-s steady contraction for BF (blue lines) and the ST (purple lines)

The experimental protocol involved isometric contractions of the knee flexors at three knee-joint angles: 0° (full extension), 45°, and 90°, as described recently (Sahinis et al. 2024). Briefly, participants laid in a prone position on an examination table, with knee-joint angle controlled by a goniometer and the hips in a neutral position (Fig. 1A). The voluntary contractions were performed with a dynamometer (MUC1, OTbiolelettronica, IT) that featured a wooden framework to stabilize the knee at selected angles, while the applied force was measured with a load cell (TF022, range 200 kg, OTbiolelettronica, IT). To maintain stability, the hip joint and trunk were secured with straps. Trials were discarded when an unacceptable amount of hip displacement was observed based on visual inspection by the investigator.

 A twin-axis goniometer (MLTS700, AD Instruments Inc., Colorado Springs, USA) was used to monitor the knee angle.

### Experimental procedure

Participants visited the laboratory on two occasions. The first visit served as a familiarization session, during which they were introduced to the laboratory environment and experimental setup and practiced submaximal isometric contractions with the knee flexors. The protocol began with a standardized warm-up, which included cycling and both static and dynamic stretching of the knee flexors. After electrode attachment on the left leg, brief submaximal isometric contractions were performed at three joint angles (0° [full extension], 45°, and 90°) in a randomized order. This was followed by three to four maximal voluntary contractions (MVCs) at each of the three joint angles, with each contraction lasting approximately 3–5 s and rest periods of 3–5 min between trials. Verbal instructions were given before each MVC, urging participants to “pull as hard as possible”. The greatest torque recorded from the MVC trials was used as a reference for subsequent submaximal isometric contractions. The torque was measured as the product of the force recorded by the load cells and the moment arm from the knee joint.

Approximately, 5 min after the MVCs, participants performed submaximal isometric contractions at target torques of 10%, 20%, 40%, and 60% MVC, in a randomized order. Each contraction involved a ramp increase from rest to the target at a fixed rate of 5% MVC/s^−1^, which was sustained for a specified duration (30 s at 10% MVC, 20 s at 20% MVC, 15 s at 40% MVC, and 10 s at 60% MVC) before a ramp decrease back to baseline (Amiridis et al. 2024; Sahinis et al. 2024). There was a permissible error margin of 5% at each target torque. Three trials were conducted for each target torque and the trial showing the lowest coefficient of variation (CoV) for torque was selected for analysis. Rest periods of ~ 1 min were given between trials. Real-time display of target and applied torques was shown on a 43-in monitor positioned at eye level, ~ 1.5 m in front of the participant (Fig. 1A). Discomfort level was monitored throughout the experiment on a scale of 0 to 10, with 0 representing no discomfort and 10 representing extreme discomfort.

### Electromyography

HD-sEMG signals were recorded from the ST and BF muscles using two-dimensional adhesive grids that comprised 64 pins (5 columns × 13 rows, gold-coated pins of 1 mm diameter and 8 mm between pins; GR08MM1305 OT bioelettronica, Italy). To ensure accurate placement over ST and to exclude the semimembranosus, longitudinal and transverse ultrasound scans were used to identify the muscles and the mediolateral borders of each muscle. The ultrasound device (SSD-3500, ALOKA, Japan) was equipped with a linear array probe operating at a frequency of 7.5 MHz and a length of 6 cm. The scans were performed at rest and during submaximal isometric contractions to visualize muscle-specific deformation and confirm that the selected region corresponded to ST. The electrode grid was placed at mid-thigh, aligned with the fascicles of each muscle where the ST has its greatest thickness (Sahinis and Kellis 2024b) and is consistently superficial to the SM (Fig. 1B). To minimize crosstalk with the BF, the grids were separated by ~ 1 cm and positioned over the muscle belly center of each muscle. The thickness of the fat tissue was measured with ultrasound for both muscles.

The skin surface was shaved, lightly abraded, and cleaned with water prior to electrode placement. To enhance the connection between the skin and electrodes, two electrode grids were positioned on the skin using disposable adhesive foam layers, with the holes filled with conductive paste (SpesMedica, Battipaglia, Italy). An elastic band (10 cm wide) was wrapped over the electrodes with slight tension to maintain electrode contact throughout the experiment. A ground electrode (dampened strap electrode) was positioned on the contralateral wrist and reference electrodes were placed on the ipsilateral patella and medial malleolus.

Both the torque and monopolar HD-sEMG signals were sampled at 2048 Hz, amplified (× 150), and band-pass filtered (10–500 Hz). These signals were recorded using a multichannel amplifier (Quattrocento, OTbiolelettronica, Italy) and digitized at 16 bits b. The OTBioLab + software (v1.5.6) was utilized to collect both the torque and HD-sEMG signals.

### Data analysis

#### Torque signal

The torque signal was low-pass filtered with a fourth-order, zero-lag Butterworth filter with a cut-off frequency of 20 Hz, after which it was converted to newton-meters (Nm). At each target torque, the steadiest 6-s interval during the isometric contraction was extracted using custom MATLAB scripts (Matlab 2024a; Mathworks, Inc.). To isolate the fluctuations in torque, the signals were band-pass filtered (second-order Butterworth) between 0.75 and 5 Hz. The normalized amplitude of the torque fluctuations was quantified as the CoV (standard deviation/mean*100%), and the absolute amplitude was measured as the standard deviation (SD).

#### EMG recordings

Offline EMG analyses were performed in MATLAB (Matlab 2024a; Mathworks, Inc.) with custom scripts. Signals were band-pass filtered from 20 to 500 Hz, and channels exhibiting low signal-to-noise ratios or artifacts were removed following visual inspection (mean ± SD: 2 ± 1 channels across knee angles). The EMG amplitude, determined as the root mean square (RMS), was derived from HD-sEMG signals by computing differential signals between adjacent electrodes along the proximo-distal axis, resulting in 59 differential signals. The rectified signals were averaged across the electrode grid during the MVC trials, and the greatest value obtained from a 500 ms moving mean was identified as the maximal EMG amplitude.

The monopolar HD-sEMG recordings were filtered with a second-order zero-phase Butterworth filter (20–500 Hz) before decomposition. HD-sEMG signals were decomposed into individual MU pulse trains using a previously validated convolutive blind-source separation algorithm with a silhouette (SIL) threshold of 0.90 (Negro et al. 2016a), implemented in the MUedit software (Avrillon et al. 2024). Identified spike trains were then manually reviewed and edited by an experienced operator following established protocols (Martinez-Valdes et al. 2023; Sahinis et al. 2024). MUs with poor signal quality (SIL < 0.90) or with discharge times exceeding 2 s were excluded from further analysis. The SIL index is a normalized measure indicating the relative height of the peaks of decomposed spike trains against baseline noise, correlating with the rate of agreement with the two-source validation method (Negro et al. 2016a). Each spike train was inspected, and interspike intervals (ISI) outside the 5–30 pps range (i.e., < 33.3 ms or > 200 ms) were excluded; short ISIs likely reflected discrimination errors or double discharges, whereas long ISIs indicated brief pauses in discharge rate. The final analysis included only those MUs that demonstrated consistent activity throughout the entire isometric contraction.

MU discharge characteristics are reported with three measures: (1) mean discharge rate (MDR) over the steadiest 6 s interval; (2) the CoV for the interspike interval (ISI) indicated discharge rate variability; and (3) neural drive was estimated by low-pass filtering the discharge train of each motor unit using a 400 ms Hanning window to produce a cumulative spike train (CST) comprising the summed binary arrays of MU discharge times. Variability in neural drive was measured as the SD of the filtered CST (SD of fCST). To ensure appropriate comparisons between muscles, target torques, and knee angles, the same number of motor units was included in the CST for each condition.

The association between torque and fCST was examined with two approaches for each muscle individually (ST and BF) and the combined fCST of both muscles. In the first approach, the correlation between torque and fCST was determined by calculating cross-correlation coefficients in overlapping 1 s epochs with a 0.25 s step (75% overlap), resulting in 21 epochs for each 6 s trial. In the second approach, the association between torque fluctuations (CoV for torque) and variability in neural drive (SD of fCST) was investigated. Using the same epoch structure (1 s windows with 75% overlap), we performed a cross-correlation analysis to examine the strength of the correlation between neural drive variability and torque fluctuations (Tvrdy et al. 2025). The mean cross-correlation coefficient was obtained by averaging the results across all epochs for both approaches. To ensure accurate temporal alignment between the signals, we optimized the signals accounting for electromechanical delay (EMD) prior to calculating the cross-correlation coefficients. The optimization involved identifying the EMD as the time lag corresponding to the peak cross-correlation between the torque and the fCST signals. This process was conducted after band-pass filtering both signals between 0.75 and 5 Hz (second-order Butterworth filter) to retain relevant signal components while removing noise. The EMD was then used to shift the signals accordingly, aligning them to account for the neuromechanical delay before performing the cross-correlation analysis (Tvrdy ﻿et ﻿al. 2025). Training has been shown to reduce neural drive variability and improve force steadiness, highlighting the functional importance of the observed associations (Lecce et al. 2025).

The level of shared synaptic input within and between the ST and BF muscles at different knee angles and target torque levels was assessed as correlations between two equally sized groups of detrended CSTs (Farina and Negro 2015). The same number of MUs was used across all trials for each condition, which depended on the minimal number of MUs available across conditions. This method is commonly used to evaluate the level of shared synaptic input within and between muscles (Farina and Negro 2015; Laine et al. 2015; Hamard et al. 2023; Sahinis et al. 2024).

#### Assessment of crosstalk and duplicates between motor units

All identified MUs were assessed to ensure no interference or overlap existed between the two muscles. The action potentials of motor units in ST and BF were compared based on spike-triggered averages. This involved calculating the average peak-to-peak amplitudes of each MU action potential across all channels in the grid, which were then compared between the grids to detect the presence of crosstalk. A larger action potential in an adjacent muscle was taken as a sign of crosstalk. Unlike traditional crosstalk analysis, which quantifies the proportion of an action potential amplitude detected in adjacent muscles, our approach ensures that each MU action potential originates primarily from an intended muscle (Hamard et al. 2023; Sahinis et al. 2024). This method provides greater specificity at the motor unit level, as it minimizes the likelihood of overlapping signals from adjacent muscles. In addition, we confirmed that no MUs were identified from electrode grids covering both the ST and BF by discarding as duplicates MUs that had ≥ 30% overlapping discharge times between the two muscles.

### Statistics

All statistical analyses were conducted using R software (version 4.3.0, Vienna, Austria). Initially, the normality of the data distribution was confirmed via quantile–quantile plots and the Kolmogorov–Smirnov test. Subsequently, a one-way, repeated-measures analysis of variance (ANOVA) was performed to assess differences in MVC torque across various knee angles (0°, 45°, and 90°). Two-way, repeated-measures ANOVAs (3 × 4) were used to examine differences in torque steadiness (CoV for torque and SD of torque) across knee angles and target torques (10%, 20%, 40%, and 60% of MVC). Another two-way, repeated-measures ANOVA (2 × 3) was used to evaluate the differences in EMG amplitude between the ST and BF across the three knee angles.

Post-hoc Tukey tests were used to identify significant differences between paired means. In addition, paired sample *t*-tests examined the differences in fat tissue thickness and the average number of identified MUs per participant between the two muscles at each target torque.

To compare MU discharge characteristics between muscles, knee angles, and target torques, linear mixed models were employed (*lme.R* function from the *nlme package*). Muscle, knee angle, and target torque were specified as fixed factors, with participant and MUs nested within participants as random factors. Similarly, linear mixed models were used to assess the differences in the correlations (r values) between muscles (ST, BF, and combined ST and BF), knee-joint angles, and target torques. The statistical significance of fixed effects was determined using ANOVA, and post-hoc pairwise comparisons with Tukey correction were conducted for significant main effects or interactions using least-squares contrasts from the lsmeans package. Effect sizes were evaluated using partial eta squared (*η*_*p*_^2^), with values interpreted as follows: < 0.01 = negligible, 0.01–0.059 = small, 0.06–0.139 = medium, and ≥ 0.14 = large effect size. Cohen’s d effect sizes were used to estimate the magnitude of differences for significant post-hoc comparisons and classified as follows: 0.2 = small, 0.5 = medium, and 0.8 = large. A *p*-value < 0.05 was considered statistically significant. The post-hoc results are reported as mean estimate and standard deviation.

## Results

The participants consistently reported a discomfort level of ≤ 4 during the experiment, indicating that the protocol was not excessively demanding.

### MVC torque

MVC torque at the three joint angles was 91.4 ± 8.6 Nm at 0º, 86.1 ± 8.9 Nm at 45º, and 80.5 ± 11.2 Nm at 90º. MVC torque was greatest at 0º (F_(2,28)_ = 9.105, *p* = 0.0009, *η*_*p*_^2^ = 0.394) compared with the other knee angles (0° vs 45°, *d* = 0.62 and 0° vs 90°, *d* = 1.09) and the least at 90° (90° vs 45°, *d* = 0.55).

### EMG amplitude during MVC

There was a significant main effect for muscle on EMG amplitude during the MVCs (F_(1,14)_ = 47.083, *p* = 0.00008, *η*_*p*_^2^ = 0.771) with the value for BF (0°: 188 ± 28 mV 45°: 170 ± 26 mV and 90°: 169 ± 25 mV) being greater than ST (0°: 170 ± 30 mV 45°: 152 ± 35 mV and 90°: 154 ± 27 mV). No other differences were observed (*p* > 0.05).

### Torque steadiness

**Fig. 2:**
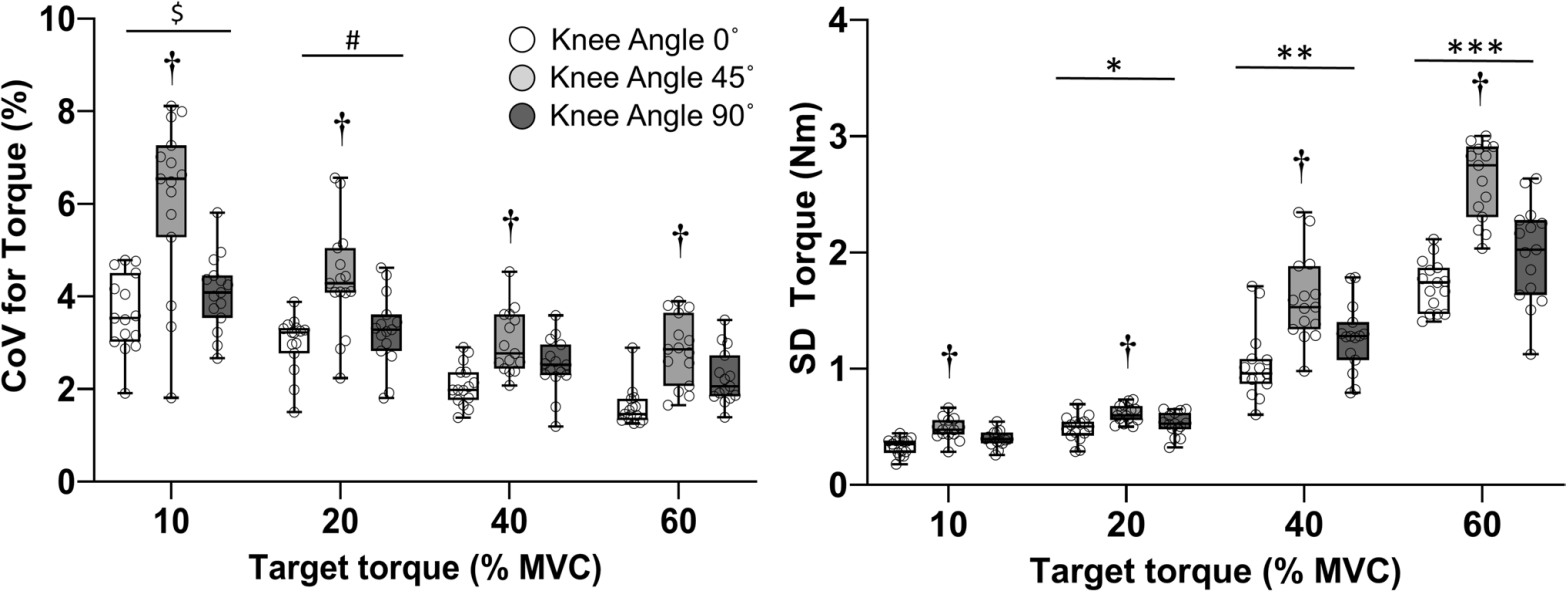
Coefficient of variation (CoV) for torque (left panel) and standard deviation (right panel) of torque during steady isometric contractions with the knee flexors at three joint angles (0° = open circles, 45° = light grey circles, 90° = dark grey circles) and four target torques: 10, 20, 40, and 60% MVC. The central line in each box represents the median with the box spanning the interquartile range (25th–75th percentile). Whiskers indicate the minimal and maximal values with each data point denoting one participant. †*p* < 0.01 relative to the other joint angles, $*p* < 0.01 relative to 20%, 40% and 60% MVC torque, #*p* < 0.01 relative to 40% and 60% MVC torque, *p < 0.01 relative to 10% MVC torque, **p < 0.01 relative to 20% MVC torque, ***p < 0.01 relative to 40% MVC torque

Figure 2 illustrates the CoV for torque and the SD of torque at three knee angles and four target torques. A significant main effect was found for knee angle (F_(2,28)_ = 40.123, *p* < 0.0001, *η*_*p*_^2^ = 0.741), with the CoV for torque being greatest at 45° (*p* < 0.0001, *d* = 0.43–1.54) and least at 0° (*p* < 0.05, *d* = 0.36–1.31). In addition, there was a significant main effect for target torque (F_(3,42)_ = 62.597, *p* < 0.0001, *η*_*p*_^2^ = 0.817) with a gradual decrease in CoV from 10 to 40% of MVC torque (p < 0.01) and then remaining constant from 40 to 60% MVC torque.

The ANOVA also revealed a significant main effect of knee angle on the SD of torque (F_(2,28)_ = 67.048, *p* < 0.001, *η*_*p*_^2^ = 0.807), with the value at 45° angle (*p* < 0.001, *d* = 1.34–1.79) being greater than the other angles. Moreover, there was a main effect for target torque (F_(3,42)_ = 224.668, *p* < 0.0001, *η*_*p*_^2^ = 0.934) with the SD for torque progressively increasing from 10 to 60% MVC torque across the three knee angles.

### Subcutaneous tissue thickness

The thickness of the subcutaneous layer did not differ (t_(14)_ = −1.43, *p* > 0.05) between the two muscles (ST: 7.4 ± 3.3 mm, BF: 7.8 ± 3.1 mm).

### Crosstalk and duplicates between motor units

The crosstalk analysis of the MU data found relatively few common motor units in the two muscles: ST = 9 (1.9%, across all knee-joint angles) at 10% MVC, 7 (1.6%) at 20% MVC, 10 (2.9%) at 40% MVC, and 6 (2.1%) at 60% MVC; BF = 8 (1.7%) at 10% MVC, 10 (2.3%) at 20% MVC, 6 (1.4%) at 40% MVC, and 5 (1.3%) at 60% MVC. The crosstalk MUs were excluded from the analysis. Moreover, 5 duplicate MUs (1.1%, across all knee-joint angles) were identified at 10% MVC, 4 (0.9%) at 20% MVC, 2 (0.6%,) at 40% MVC, and 3 (1.0%) at the 60% MVC. These MUs were discarded from the analysis.

### Motor unit decomposition

The number of identified MUs across participants for the four target torques and the three knee angles was 1517 for ST and 1683 for BF (Supplementary File 1). The median (25–75% percentile) number of identified MUs per participant for ST and BF, respectively, was 10 (9–11) and 11 (10–12) at 10% of MVC, 9 (9–10) and 10 (9–11) at 20% of MVC, 8 (7–9) and 8 (8–10) at 40% of MVC, 7 (6–7) and 8 (7–9) at 60% of MVC. The number of identified MUs was not significantly different between the two muscles at any of the target torques (*p* > 0.05 in all cases).

### Mean discharge rate

**Fig. 3:**
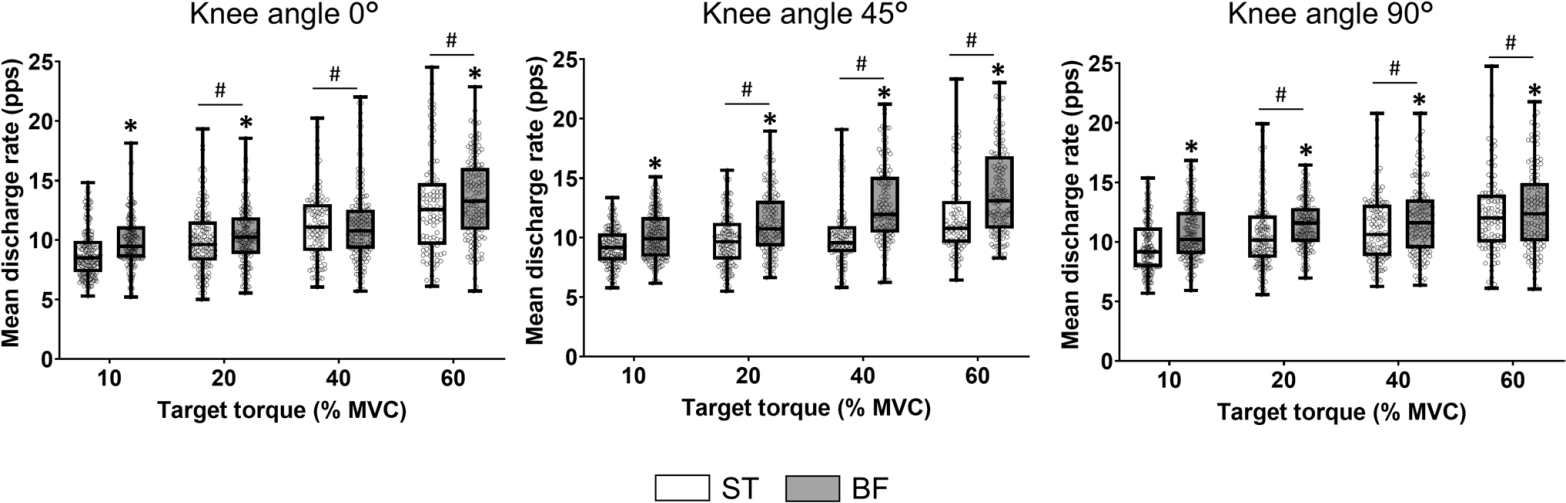
Mean discharge rate of MUs in semitendinosus (ST) (open box) and biceps femoris (BF) (filled box) during steady contractions with the knee flexors at four target torques (10, 20, 40, and 60% MVC) across three knee angles (0º = long muscle length, 45º = intermediate muscle length and 90º = short muscle length). Boxplot details as described in Fig. 2. Each data point corresponding to one motor unit. *p < 0.01 relative to ST, #*p* < 0.01 compared with lower target torques

Recruitment thresholds were distributed similarly for ST and BF, respectively, across the target torques (collapsed across knee angles): 10% = 1.1–9.4% MVC and 1.2–9.3% MVC; 20% = 1.1–19.1% and 1.2–19.3% MVC; 40% = 2.0–38.1% and 2.1–38.3% MVC, and 60% = 2.9–56.6% and 3.1–56.8% MVC. There was a significant main effect of MDR (Fig. 3) for muscle (*F* = 126.899, *p* < 0.00001, *η*_*p*_^2^ = 0.038), with the value being greater for BF [11.6 ± 1.2 pps] than ST [10.6 ± 1.3 pps; *d* = 0.3–1.32]. There was also a significant main effect of knee angle (*F* = 7.347, *p* = 0.0006, *η*_*p*_^2^ = 0.004), with MDR being greater at 90° [11.3 ± 1.1 pps] than at 0° [10.9 ± 1.4 pps; *p* < 0.001, *d* = 0.22–0.37]. Moreover, MDR increased progressively with target torque from 10% [9.7 ± 3.7 pps] to 60% [12.7 ± 0.7 pps] of MVC torque (*F* = 202.447, *p* < 0.00001, *d* = 0.20–1.33).

### Discharge variability (CoV for ISI)

**Fig. 4:**
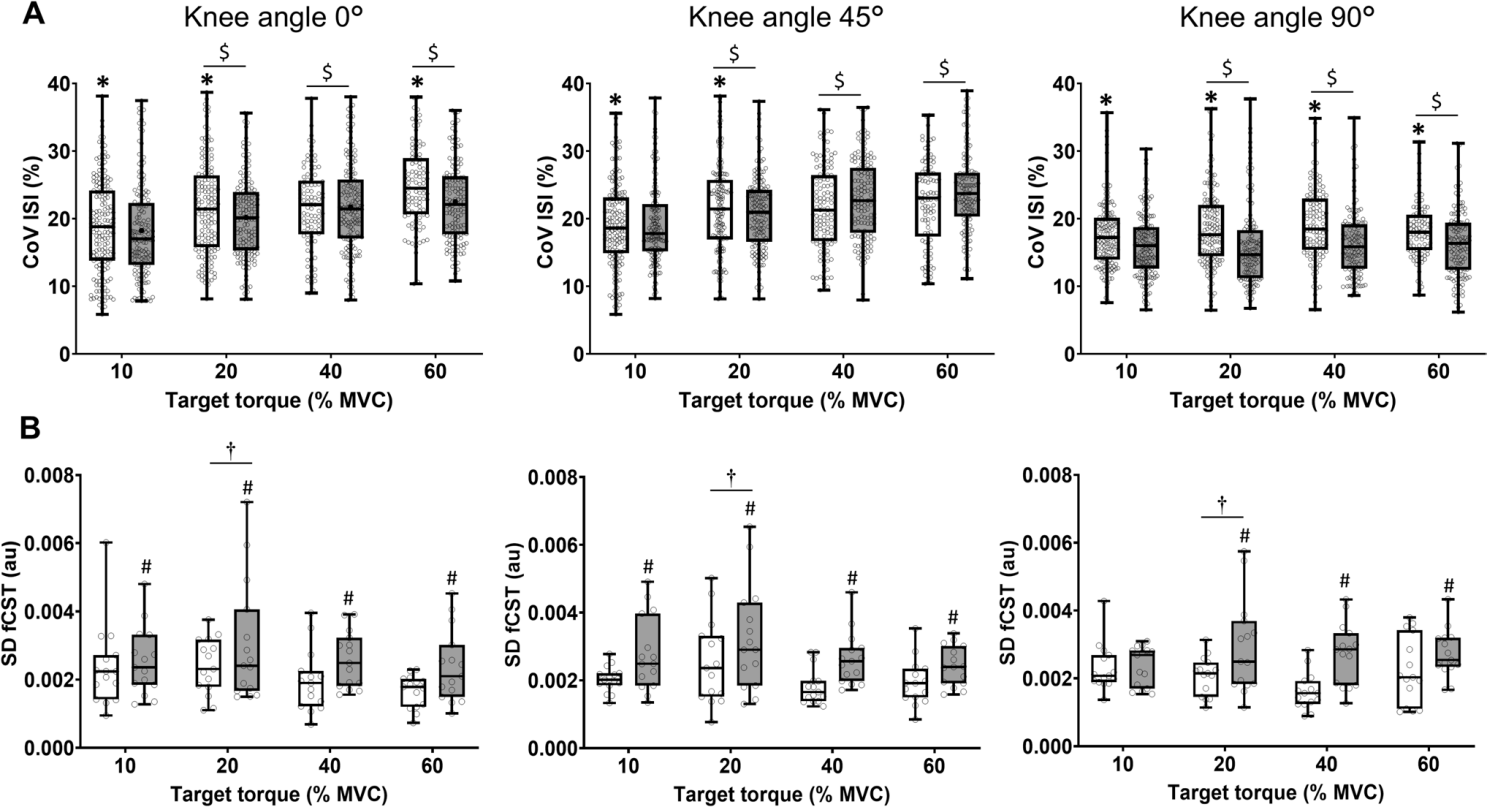
**A** Coefficient of variation (CoV) for interspike interval (ISI) and **B** standard deviation (SD) of the filtered cumulative spike train (fCST) for motor units in semitendinosus (ST) (open box) and biceps femoris (BF) (filled box) during steady contractions with the knee flexors at four target torques (10, 20, 40, and 60% MVC) across three knee angles (0º = long muscle length, 45º = intermediate muscle length and 90º = short muscle length). Boxplot details as described in Fig. 2. Each data point corresponding to the CoV for ISI for one motor unit (upper panels) or the average value for SD of fCST of one participant (lower panels). *p < 0.01 relative to BF, #*p* < 0.01 relative to ST, $*p* < 0.01 relative to lower target torques, †*p* < 0.01 relative to other target torques

Figure 4 displays the CoV for ISI at each knee angle for the four target torques. There was a significant muscle × angle (*F* = 20.669, *p* < 0.00001, *η*_*p*_^2^ = 0.013) and angle × target torque interaction (*F* = 9.016, *p* < 0.00001, *η*_*p*_^2^ = 0.017) for CoV of ISI. Post-hoc analysis indicated that the CoV for ISI was greater for ST [20.4 ± 2.2%] than BF [19.5 ± 2.9%] across knee-joint angles (*p* = 0.0001–0.000002, *d* = 0.33–0.884). In addition, the CoV for ISI was less at 45° [17.2 ± 1.2%] than at other knee-joint angles [0°: 21.2 ± 2.1% and 90°: 21.5 ± 1.8%; *p* = 0.01–0.0001, *d* = 0.48–1.19]. The CoV for ISI increased with target torque from 10% [18.1 ± 1.2%] to 60% [21.5 ± 3.5%] of MVC (*F* = 51.196, *p* < 0.00001, *d* = 0.23–1.00).

### Variability in neural drive (SD of fCST)

Figure 4 plots the average SD of fCST across the four target torques for each knee angle. There was a significant main effect of muscle (*F* = 34.745, *p* = 0.00001, *η*_*p*_^2^ = 0.097) but not knee-joint angle (*F* = 0.436, *p* = 0.646, *η*_*p*_^2^ = 0.0002). The SD of fCST was less (*p* = 0.01–0.0002, d = 0.49–0.93) for the ST [0.002 ± 0.0007 au] than the BF [0.003 ± 0.0002 au]. The SD of fCST increased between 10% [0.003 ± 0.0008 au] and 20% [0.004 ± 0.0003 au] of MVC and then decreased between 20 and 40% [0.002 ± 0.0005 au] of MVC and then remained stable (60%: 0.002 ± 0.0003 au; *F* = 7.002, *p* = 0.0001, *d* = 0.46–1.2).

### Correlation between torque and fCST

**Fig. 5:**
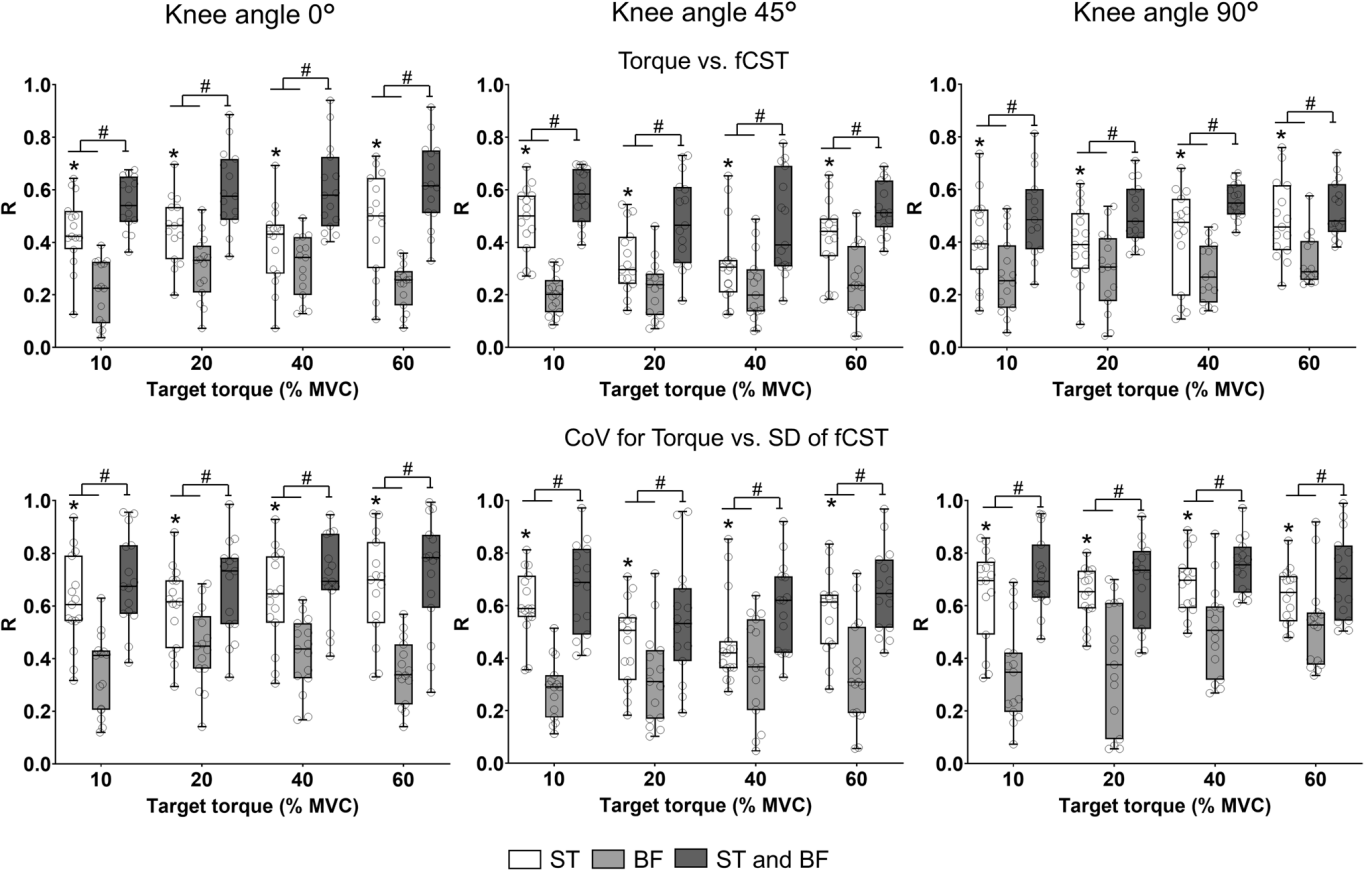
Correlation analysis between the torque and filtered cumulative spike train (fCST) (upper panels) and coefficient of variation (CoV) for torque and the standard deviation (SD) of the fCST (lower panels). The graphs plot data for semitendinosus (ST) (open box), biceps femoris (BF) individually (light grey box), and when combined (dark grey box) at the four target torques (10, 20, 40, and 60% MVC) across the three knee angles (0º = long muscle length, 45º = intermediate muscle length and 90º = short muscle length). Boxplot details as described in Fig. 2. Each data point corresponding to one participant. The correlation values were greater for ST than BF. *p < 0.01 relative to BF, #*p* < 0.01 relative to individual values for ST and BF

The upper panels in Fig. 5 display the average correlations between torque and neural drive (fCST) for each muscle and for the combined fCST of both muscles during steady contractions across the three knee-joint angles. A significant interaction was found between muscle × target torque (*F* = 2.765, *p* = 0.008, *η*_*p*_^2^ = 0.034), as well as between knee angle × target torque (*F* = 3.443, *p* = 0.0013, *η*_*p*_^2^ = 0.041). Post-hoc tests indicated that the correlation between torque and fCST was greatest for the combined fCST (0.47 ± 0.07, *p* = 0.0001–0.005, *d* = 0.39–0.98) than for either ST [0.41 ± 0.05] or BF [0.28 ± 0.05] (*p* = 0.0004–0.007, *d* = 0.54–1.19). However, the correlations for ST were significantly greater than those for BF. Furthermore, the correlation values were less at 45° knee angle [0.35 ± 0.10] than the other two knee angles (0°: 0.41 ± 0.09 and 90°: 0.41 ± 0.08; *p* = 0.0002–0.007, *d* = 0.58–1.27).

### Correlation between CoV for torque and SD of fCST

The lower panels in Fig. 5 present the average correlations between torque variability (CoV for torque) and neural-drive variability (SD of fCST) for each muscle and for the combined fCST during steady contractions at different knee-joint angles. There was a significant muscle × target torque interaction (*F* = 5.512, *p* = 0.0012, *η*_*p*_^2^ = 0.047) and knee angle × target torque (*F* = 3.476, *p* = 0.0016, *η*_*p*_^2^ = 0.057). Post-hoc tests revealed that the correlation between the CoV of torque and the SD of fCST was greatest for the combined fCST of both muscles (0.66 ± 0.07; *p* = 0.0002–0.004, *d* = 0.44–1.27) than for either ST [0.64 ± 0.12] or BF [0.38 ± 0.08] (*p* = 0.0001–0.001, *d* = 0.53–1.31). However, the correlations for ST were significantly greater than those for BF. In addition, correlation values were less at 45° knee angle [0.51 ± 0.17] than the other two knee angles (0°: 0.56 ± 0.13 and 90°: 0.62 ± 0.15; *p* = 0.0001–0.004, *d* = 0.51–1.06).

### Correlation for fCST within and between muscles

**Fig. 6:**
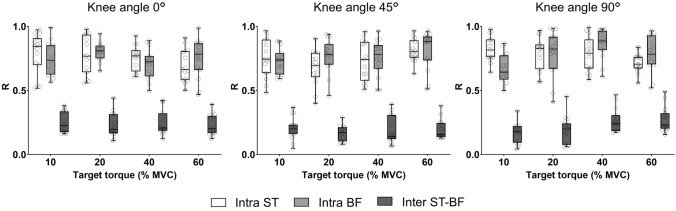
Correlation analysis between the filtered cumulative spike train (fCST) for identified motor units from semitendinosus (open box), biceps femoris (light grey box), and semitendinosus vs biceps femoris muscles (dark grey box) at the four target torques (10, 20, 40, and 60% MVC) across the three knee angles (0º = long muscle length, 45º = intermediate muscle length and 90º = short muscle length). Boxplot details as described in Fig. 2. Each data point corresponding to one participant. There were high correlation values for the fCSTs within each muscle, but not between the two muscles

There was a strong correlation between the fCSTs for the MUs within each muscle collapsed across the knee angles (ST: 10%, 0.49–0.97; 20%, 0.55–0.95; 40%, 0.51–0.93; 60%, 0.50–0.91 and BF: 10%, 0.55–0.95; 20%, 0.52–0.94; 40%, 0.50–0.96; 60%, 0.48–0.96). In contrast, the correlation collapsed across knee-joint angles between muscles was much weaker (10%: 0.03–0.23; 20%: 0.04–0.21; 40%: 0.08–0.31; 60%: 0.01–0.23) (Fig. 6).

## Discussion

The main finding of our study was that MU discharge characteristics and neural drive differed between two hamstrings muscles (ST and BF) during submaximal isometric contractions across three knee-joint angles. Notably, BF exhibited greater MDR, variability in neural drive (SD of fCST), and EMG amplitude during MVC compared with ST. Conversely, the ST displayed greater variability in discharge times (CoV for ISI) and a stronger correlation between SD of fCST and CoV for torque than BF.

### Differences between the ST and BF

We observed distinct neural drive characteristics for two synergistic hamstring muscles (ST and BF), which is consistent with previous reports (Kirk and Rice 2017; Kirk et al. 2018). It has been shown, for example, that gastrocnemius medialis and gastrocnemius lateralis operate relatively independently during both plantar flexion and knee flexion (Hamard et al. 2023), as well as during plantar flexion tasks at different ankle angles (Levine et al. 2023). The specialized control of synergistic muscle groups likely enhances the adaptability to various mechanical and physiological demands (Farina and Negro 2015; Hug et al. 2023).

The distinct neural drives to BF and ST reflect differences in the modulation of the motor neuron pools by synaptic inputs from premotor interneurons. The specificity is at least partially due to differences in fiber-type composition (Garrett et al. 1984), fascicle architecture (Kellis et al. 2012; Kellis 2018), and the mechanical properties of each muscle–tendon unit (Kellis 2016; Kellis and Sahinis 2022; Sahinis and Kellis 2024a). For instance, BF has a greater physiological cross-sectional area and pennation angle compared with ST, which enhances its force-generation capacity relative to other hamstrings muscles (Kellis et al. 2012; Kellis 2018). Conversely, ST has nearly twice the normalized fascicle-to-muscle length ratio, providing a relative advantage at longer muscle lengths for force production compared with BF (Kellis et al. 2012; Kellis 2018). In addition, BF experiences less strain and elongation of its tendon/aponeurosis than ST, particularly during strong contractions, which underscores the different roles of these two synergists during knee flexion (Kellis 2018). When combined with prior research on neural drive differences within the ST (Sahinis et al. 2024), the current findings suggest that BF and ST are likely controlled by more than one independent source of synaptic input. Given that BF and ST contribute to both knee and hip movements, they may require specialized neural control to coordinate flexion, extension, and stabilization across joints.

Recent work suggests that synergists, such as vastus lateralis and vastus medialis, are controlled by flexible neural modules (Del Vecchio et al. 2023; Weinman et al. 2024). It has been proposed that such flexibility in the control of synergistic muscle groups may enhance the ability to accommodate varying mechanical demands thereby optimizing force production and reducing injury risk (Hug et al. 2023). This possibility is particularly crucial for the hamstrings, which play key roles in knee flexion, hip extension, and pelvic stabilization during activities like sprinting. For example, although both ST and BF exhibit high EMG amplitudes from the middle of the swing phase to the start of the stance phase during running (Higashihara et al. 2010; Schache et al. 2013), BF displays greater EMG amplitudes during the middle-to-late swing phase, whereas the greatest EMG amplitude in ST occurs during the terminal swing phase (Higashihara et al. 2010).

Our findings on MU discharge characteristics contrast with a previous report (Kirk and Rice 2017) of greater MDR for the medial hamstrings (ST and semimembranosus) than BF. This discrepancy may arise from methodological differences, such as variations in electrode placement or the position of the participant (hip flexed at 100°) (Kirk and Rice 2017). Moreover, the limitations of needle EMG recordings, including the detection of relatively few MUs, cannot characterize MU activity at a population level. In addition, both muscles have compartmentalized architecture with partially independent innervation (Tubbs et al. 2006; Kellis 2018; Sahinis et al. 2025b), which may lead to regional differences in activation (Hegyi et al. 2019; Sahinis et al. 2024). In the present study, the electrode grids were likely positioned over the distal region of each muscle, which may partially explain discrepancies with prior work.

### ST influences torque variability

The greater correlation between torque and fCST  observed in ST than BF during submaximal isometric contractions underscores the differential contributions of these muscles to torque control during this task (Fig. 5). Several factors may explain the stronger correlation for ST. For example, ST and BF differ in their moment arms and attachment sites around the knee, resulting in different lines of action (Kellis and Blazevich 2022). The longer moment arm of the ST at the knee joint may enhance its role in stabilizing the knee joint and controlling an applied force (Kellis and Blazevich 2022). In contrast, BF has a shorter moment arm but larger PCSA, which suggests that it may be more involved in generating force and power rather than controlling an applied force (Kellis and Blazevich 2022). In addition, the difference in tendon/aponeurosis strain between the ST and BF during knee flexion, with the ST showing approximately 10–12% greater displacement (Kellis 2016), may lessen the capacity of BF to dampen torque fluctuations.

Quantification of force fluctuations during steady muscle contractions reveals important insights into the variability of control signals transmitted from motor neurons, which are essential for precise motor actions (Negro et al. 2016b; Enoka and Farina 2021). This variability provides an estimate of the noisiness of the control signal, as measured by the CoV for ISI and the SD of fCST, respectively (Enoka and Farina 2021). Although motor neurons individually exhibit nonlinear input–output relations, the collective activity of many neurons tends to linearize these interactions, effectively reducing the influence of synaptic noise and simplifying the overall control of force (Farina et al. 2016). Although force steadiness (CoV for force) differed across knee-joint angles, there were no accompanying changes in the SD of fCST (Sahinis et al. 2024). This dissociation reflects the contribution of several muscles to the overall knee-flexion force, each possesing unique anatomical and biomechanical properties , whereas the SD of fCST was derived from individual muscles. This interpretation is consistent with the greater correlation observed when the summed SD of fCST for the two muscles was cross-correlated with the torque fluctuations (Fig. 5). In a simpler action involving a single hand muscle, for example, a large proportion of the variability in force fluctuations during submaximal isometric contractions can be explained by the variability in MU activity (Negro et al. 2009; Tvrdy et al. 2025).

### Influence of muscle length

MVC torque was greatest at longer muscle lengths, consistent with findings from previous studies (Kellis and Blazevich 2022; Sahinis et al. 2024). In addition, force variability (CoV for torque and SD of torque) was greater at intermediate muscle lengths across all force targets, aligning with our recent study (Sahinis et al. 2024). However, the greater torque variability at intermediate muscle lengths cannot be fully explained by differences in motor unit discharge characteristics, as neural drive fluctuations of the individual muscles did not differ significantly across joint angles. Similarly, the CoV for ISI values were least at the intermediate muscle length, which was not associated with the greater torque fluctuations at this knee-joint angle (Fig. 4). However, the CoV for ISI, which reflects synaptic noise, only influences the CoV for force at low forces (Tvrdy et al. 2025).

Therefore, the observed increase in torque variability at intermediate lengths is unlikely to be solely due to neural factors. The greater force fluctuations at intermediate muscle lengths appear to indicate changes in the relative contributions of the involved muscles to the applied torque (Kellis 2018). For example, BF and ST function over different operating ranges on the sarcomere length-tension curve, which likely influences the force produced by each muscle (Kellis and Blazevich 2022). In addition to shifts in muscle contributions, off-direction forces may alter load distribution between the synergistic muscles (Nelson and Roberts 2008), further contributing to the observed fluctuations in torque steadiness.

The MDR of MUs in ST and BF was greater at a short muscle length (90°) than at the long muscle length (0° = full extension), consistent with findings from previous studies (Kirk and Rice 2017; Sahinis et al. 2024). For example, Sahinis et al. (2024) reported greater MDR in both compartments of the ST muscle at shorter lengths relative to longer lengths. Although the target torques were set relative to the MVC value at each knee-joint angle, these findings indicate that the same relative target required greater MDRs at shorter muscle lengths. This adjustment is likely a consequence of the decrease in twitch durations and less summation of motor unit forces at short muscle lengths (Marsh et al. 1981; Bigland-Ritchie et al. 1992).

### Future perspectives

Our findings have important clinical applications, especially for targeted training and rehabilitation of ST and BF muscles. Given the distinct neural drive and different roles of these muscles in the control of force, training programs should be tailored to the properties of each muscle. Given the ST’s role in force modulation, it may benefit from exercises that emphasize precision and stability, such as slow and controlled movements like the Nordic hamstring curl (Kellis and Blazevich 2022; Sahinis et al. 2025a). These exercises can enhance the ability of the muscle to control force. In contrast, BF has a greater force capacity and should be targeted with exercises that emphasize strength and power, which is crucial for injury prevention (Kellis and Blazevich 2022; Sahinis et al. 2025).

### Limitations

This study has several limitations that warrant consideration. First, there is significant variation in muscle architecture and innervation along the length of ST and BF, which makes it possible that our surface EMG recordings (see methods) likely captured MU activity in the superficial parts of each muscle. Second, the finding of relatively independent control of ST and BF should not be generalized beyond submaximal isometric contractions, such as dynamic locomotor tasks. Third, although B-mode ultrasound was used to identify the anatomical boundaries of the muscles prior to electrode placement, some uncertainty regarding recording selectivity remains. Despite careful placement over the ST and efforts to avoid overlap with the semimembranosus and BF, a degree of signal contamination from adjacent muscles cannot be entirely ruled out due to the inherent limitations of surface EMG. However, Kingston and Acker (2018) showed that surface EMG did not reliably capture semimembranosus activity compared to fine wire EMG, likely due to its deeper anatomical location, supporting the likelihood that our recordings primarily reflected ST activity. Although we found that crosstalk between the two muscles (ST vs BF) was minimal, with fewer than 1% of units identified as ‘crosstalk’ units, this potential source of error must be acknowledged (Avrillon et al. 2021; Hamard et al. 2023). Fourth, the excl﻿usion of females, largely due to challenges in identifying enough MUs, limits the generalizability of our findings across sexes. Fifth, by concentrating on changes in knee-joint angle, we have neglected the impact of hip-joint angle on motor unit activity, which may be important because ST and BF are biarticular muscles that contribute to actions about both hip and knee joints. Sixth, we were unable to track the MUs across knee angles due to changes in the shapes of the MU action potentials.

## Conclusion

There are distinct differences in MU discharge characteristics and neural control of the ST and BF muscles during submaximal isometric contractions. These findings suggest that each muscle receives unique synaptic input, leading to individualized contributions to the net knee-flexor torque. One functional consequence of this difference was that the ability to apply a steady torque during submaximal isometric contractions was more strongly associated with the variability in neural drive for ST than for BF. These differences likely reflect the distinct mechanical demands placed on each muscle, enhancing their ability to contribute effectively to movement capabilities.

## Supplementary Information

Below is the link to the electronic supplementary material.

Supplementary file1 (DOCX 15 KB)

## Data Availability

The data supporting the findings of this study are available upon reasonable request from the corresponding author.
