# Supplementary material for: Independent neural drives and distinct motor unit discharge characteristics in hamstring muscles during isometric knee flexion
Source: Eur J Appl Physiol. 2025 Sep 4;126(2):839–52. doi: 10.1007/s00421-025-05953-5 (PMC12948821; doi:10.1007/s00421-025-05953-5)
Supplement: Supplementary file 1 — Supplementary file1 (DOCX 15 KB) [file 421_2025_5953_MOESM1_ESM.docx]

**Supplementary Material 1:** Median (25% - 75% IQR) of the identified motor units for the proximal and distal compartments of semitendinosus across the three joint angles and four target forces.

|  | Target Force | | | |
| --- | --- | --- | --- | --- |
|  | 10% MVC | 20% MVC | 40% MVC | 60% MVC |
|  | Knee angle 0° | | | |
| BF | 8 (7-10.25) | 8.5 (7.75-10) | 7.5 (6-8.25) | 5.5 (5-6) |
| ST | 9 (8-10) | 9 (8-11) | 7.5 (6-9) | 5 (5-6.25) |
|  | Knee angle 45° | | | |
| BF | 8 (7-9.25) | 8 (6.75-10) | 7 (6-8) | 5 (4-6) |
| ST | 8.5 (8-9.25) | 8.5 (7-10) | 7.5 (6-8) | 5 (4.75-6.25) |
|  | Knee angle 90° | | | |
| BF | 9.5 (7-10) | 8.5 (6-10.25) | 7.5 (6-9) | 5 (4-6) |
| ST | 9 (7.5-9.25) | 8 (7-9) | 7 (6-9) | 5 (4-6) |
